# Efficacy and Tolerability of Viloxazine Extended-Release for Attention-Deficit/Hyperactivity Disorder in Adolescents and Adults: A Systematic Review With Qualitative Synthesis of All Studies and Meta-Analysis of Adolescent Data

**DOI:** 10.7759/cureus.105176

**Published:** 2026-03-13

**Authors:** Savannah R Chapman, Christian Roberti, Alex Abouafech, Margaret Mueller

**Affiliations:** 1 School of Medicine, Lake Erie College of Osteopathic Medicine, Bradenton, USA; 2 Emergency Department, Sturdy Memorial Hospital, Attleboro, USA

**Keywords:** adolescent psychiatry, attention-deficit hyperactivity disorder (adhd), non-stimulant adhd treatment, qelbree, systematic review and meta-analysis, viloxazine extended-release

## Abstract

Viloxazine extended-release (ER) is a non-stimulant medication approved for the treatment of attention-deficit hyperactivity disorder (ADHD) in pediatric and adult populations. While numerous randomized controlled trials (RCTs) have demonstrated its efficacy in young populations, evidence specific to adolescents remains limited, and even fewer data on adults exist. This systematic review and meta-analysis evaluates the efficacy and tolerability of viloxazine ER in adolescents and adults through a qualitative synthesis of all available studies and a meta-analysis of adolescent data. A literature search was conducted in January 2026 using PubMed, the Cochrane Library, Google Scholar, and ClinicalTrials.gov. Eligible studies included RCTs evaluating the efficacy of viloxazine ER in adolescent or adult populations diagnosed with ADHD according to the* Diagnostic and Statistical Manual of Mental Disorders, 5th Edition (DSM-5)* criteria. Three studies were included in the review. Quantitative meta-analysis was performed when at least two studies were available within the same population. Effect sizes were calculated as standardized mean differences using Hedges’ g under a random-effects model. Adult outcomes were summarized descriptively due to limited data. Risk of bias was assessed using the National Institute of Health (NIH) Study Quality Assessment Tool. Safety outcomes were assessed qualitatively. Three RCTs met the inclusion criteria. Two adolescent trials (n = 593) were pooled for meta-analysis. Viloxazine ER was associated with a statistically significant reduction in ADHD symptom severity in adolescents compared with placebo (Hedges’ g =-0.33, 95% CI -0.50 to -0.16) with no observed heterogeneity (I² = 0%). One adult RCT demonstrated statistically significant improvement in ADHD symptoms compared with placebo. Viloxazine ER was generally well tolerated with low discontinuation rates and mild adverse events. Viloxazine ER is associated with modest and consistent but statistically significant efficacy in reducing ADHD symptoms among adolescents, with a favorable tolerability profile. Although adult evidence is limited, the available data suggest that viloxazine ER is beneficial to this population as well. The findings overall support viloxazine ER as an effective non-stimulant pharmacotherapy option for adolescents with ADHD, particularly so when stimulant therapy is not tolerated. Further research should prioritize long-term outcomes and efficacy in adult populations.

## Introduction and background

Attention-deficit hyperactivity disorder (ADHD) is a neuro-developmental disorder characterized by persistent patterns of inattention, impulsivity, and/or hyperactivity that interfere with daily functioning [1]. Diagnostic criteria require that symptoms be present for at least six months, occur in more than one setting, and have an onset before the age of 12 years [2]. The estimated prevalence of ADHD is approximately 5-7% in children and adolescents and 2-5% in adults [1-3]. Although ADHD is often thought of as a childhood disorder, symptoms persist into adulthood in more than two-thirds of affected individuals [3]. Untreated ADHD is associated with significant functional impairment, including reduced quality of life, increased rates of psychiatric co-morbidities, and increased mortality rates, highlighting the importance of continued and lifelong treatment when present in adulthood [2]. 

Pharmacologic treatment, in conjunction with psychosocial interventions, remains the cornerstone of ADHD treatment. Stimulant medications remain the gold standard treatment and are effective in over 90% of patients [2]. Non-stimulant medications play an important role in individuals who do not tolerate stimulants or experience adverse effects. Non-stimulant agents such as atomoxetine, guanfacine, and clonidine offer advantages including lower abuse potential and reduced cardiovascular complications [1,4]. 

Viloxazine extended-release (ER) (Qelbree) is an emerging non-stimulant medication approved in 2021 for pediatric patients and in 2022 for adults for the treatment of ADHD [5]. Originally developed as an immediate-release antidepressant, viloxazine was later repurposed into an ER preparation for ADHD management [5,6]. The therapeutic effects of viloxazine are attributed to its inhibition of norepinephrine reuptake and modulation of the serotonergic system, mechanisms that result in improved attention and behavioral regulation [6]. 

Multiple randomized controlled trials (RCTs) have demonstrated the efficacy of viloxazine ER in pediatric and adolescent populations broadly, consistently showing greater improvement in ADHD symptoms vs placebo. However, fewer analyses have focused specifically on adolescents, and evidence supporting its use in adults remains limited. 

Adolescence represents a distinct developmental period characterized by unique neurobiological, environmental, and psychosocial influences that may alter symptom presentation and treatment response compared with younger children and adults [7]. Thus, synthesizing evidence specific to this population is clinically relevant. 

The systematic review and meta-analysis presented here aims to evaluate the efficacy of viloxazine ER for the treatment of ADHD in adolescent populations by quantitatively synthesizing data from RCTs. When sufficient data were available, a meta-analysis was performed to estimate pooled treatment effects. Adult trial data were included in a qualitative synthesis but were not pooled due to the limited number of eligible studies. 

Tolerability and safety are critical considerations in pharmacotherapy, particularly for medications intended for long-term usage. This review also summarizes reported adverse events, discontinuation rates, side-effect profiles, and overall tolerability of viloxazine ER across the included studies. Safety and tolerability data from both adolescent and adult populations were reviewed qualitatively to contextualize efficacy findings. 

By clarifying the magnitude of viloxazine ER’s treatment effect in adolescents with ADHD, this study seeks to guide clinical decision-making and identify populations in which further research is needed. This analysis evaluates changes in ADHD symptom severity from baseline using the ADHD Rating Scale-5 (ADHD-RS-5) in adolescents and Adult ADHD Investigator Symptom Rating Scale (AISRS) in adults, comparing viloxazine ER with placebo [8,9]. These findings aim to further define the clinical value of viloxazine ER as a non-stimulant treatment option across adolescent and adult populations.

## Review

Materials and methods

Reporting

The Preferred Reporting Items for Systematic Reviews and Meta-Analyses (PRISMA) criteria were followed for this review [10]. 

Research Question

How effective and tolerable is viloxazine ER for the treatment of ADHD across adolescent and adult populations?

Inclusion Criteria

Studies meeting all predefined inclusion criteria were eligible for this meta-analysis. Included studies reported ADHD outcomes following treatment with viloxazine ER in adolescent (12-17 years) or adult (18-64 years) populations. Only RCTs were eligible for inclusion. Eligible studies required participants to be diagnosed with ADHD in accordance with the *Diagnostic and Statistical Manual of Mental Disorders, Fifth Edition* (DSM-5) [11]. 

Exclusion Criteria

Excluded study designs included systematic reviews, retrospective studies, cross-sectional studies, qualitative studies, case reports, case series, and non-RCTs. Studies published in non-English languages were excluded due to limitations in ensuring accurate translation. Studies not evaluating ADHD symptoms following viloxazine ER use in adolescents or adults were not considered.

Search Strategy

A comprehensive literature search was conducted through January 2026 using PubMed, The Cochrane Library, Google Scholar, and Clinicaltrials.gov. Given the high volume for Google Scholar, results were sorted by relevance, and the first 200 records were screened. Search terms included “attention deficit hyperactivity disorder,” “ADHD,” “attention deficit,” “viloxazine,” “viloxazine extended-release,” “Qelbree,” ”SPN-812,” “RCT,” and “randomized controlled trial.” This combined literature search yielded 315 records, which were subsequently screened based on the predefined inclusion and exclusion criteria. 

Data Sources

Manual sources of pertinent studies were also searched to identify studies not captured in our initial search in January 2026. 

Study Selection

A total of 315 records were screened for eligibility based on predefined inclusion and exclusion criteria as described above. Of these, 265 records were excluded at the title and abstract level due to non-RCT design, pediatric-only population, absence of ADHD-specific outcome measures, or inclusion of non-ADHD populations. A total of 32 records were identified as duplicate records and removed. Around 12 records were marked as ineligible by automation tools. This resulted in six full-text articles being assessed for eligibility. Three of these full-text articles were additionally excluded because they enrolled exclusively pediatric populations without adolescent data. Ultimately, three full-text articles were included in our final data analysis (n = 3). No disagreements occurred between reviewers (S.C. and C.R.). 

Data Extraction

Data pertaining to study title, author, publication year, sample size, study design, change in ADHD-RS-5 or AISRS scores, standard deviation, adverse events, and discontinuation rates were extracted from each study. 

Outcome Measures

The primary outcome measure in adolescent studies was changed from baseline in ADHD symptom severity over a six-week period, measured using the ADHD-RS-5. In adult studies, symptom severity was likewise measured over a six-week period and assessed with the AISRS. Mean change scores from baseline to the end of the study were extracted for analysis. 

Quality Assessment

To minimize bias, only RCTs were included in this study. Risk of bias was assessed utilizing the National Institute of Health (NIH) Study Quality Assessment Tool due to its applicability to RCT studies [12]. Funnel plot analysis was not performed due to the small number of included studies, as such an assessment would be unreliable [13]. 

Statistical Analysis

All eligible randomized controlled trials were included in the qualitative synthesis. Quantitative meta-analysis was performed only when at least two studies were available within the same population. As only one adult trial met the inclusion criteria, adult outcomes were summarized descriptively and were not pooled. 

Effect sizes were calculated as standardized mean differences using Hedges’ g to account for small sample bias. Standard errors were derived from the reported 95% CI when not provided. A random-effects meta-analysis was performed using restricted maximum likelihood. All statistical analyses were conducted using a random-effects model.

Assessment of Results

Pooled effect estimates are presented with corresponding 95% CIs. Forest plots were generated to visually display individual study effect sizes and the pooled treatment effect. Statistical heterogeneity was assessed using Cochran’s Q test and the I² statistic. Formal assessment of publication bias was not completed due to the limited number of included studies. 

Results

Literature Search

**Figure 1:**
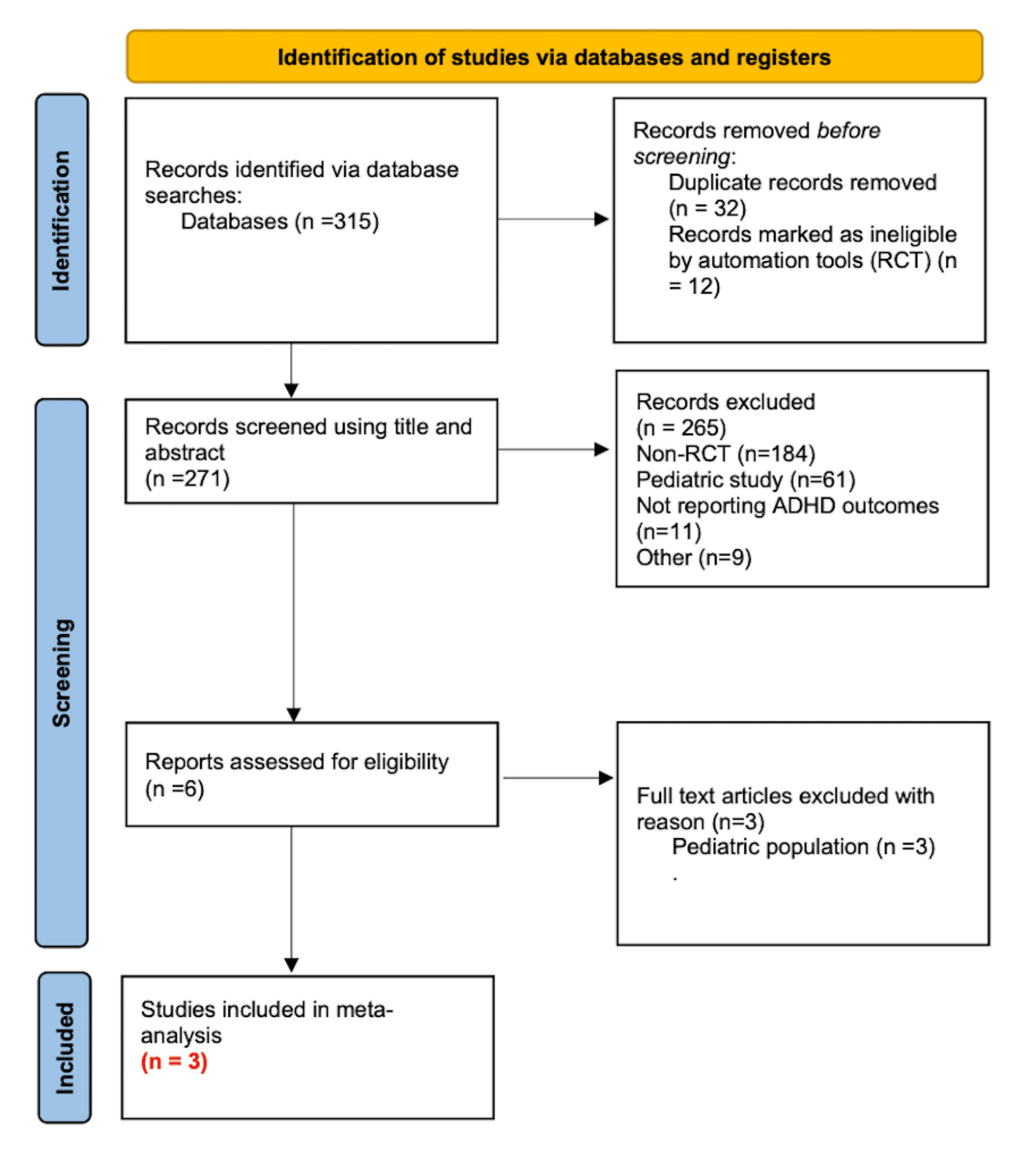
A PRISMA flowchart summary of methodology n: number; RCT: randomized controlled trial; PRISMA: Preferred Reporting Items for Systematic Reviews and Meta-Analyses

Literature search initially identified 315 records. Three studies were identified as meeting predefined inclusion criteria and included in our analysis (Figure 1) [14-16].

*Characteristics of Included Studies* 

The characteristics of the included studies are summarized in Table 1 [14-16].

**Table 1:** Summary of the literature search and study characteristics included in the data analysis USA: United States of America; ADHD: attention-deficit hyperactivity disorder; n: number; ADHD-RS-5: ADHD Rating Scale-5; AISRS: Adult ADHD Investigator Symptom Rating Scale

Study	Location	Total sample size (n)	Viloxazine receiving group size (n)	Placebo group size (n)	Study design	Population/age range	Intervention (dose)	Duration	Primary outcomes measured	Key efficacy results
Nasser et al. (2022) [14]	USA	374	190	184	Phase III, randomized, double-blind, placebo-controlled, two-arm trial	Adults (18-65 years)	200-600 mg ER	Six weeks	AISRS	Significant improvement vs placebo
Nasser et al. (2021) [15]	USA	301	197	104	Phase III, randomized, double-blind, placebo-controlled, two-arm trial	Adolescents (12-17 years)	200-400 mg ER	Six weeks	ADHD-RS-5	Significant improvement vs placebo
Nasser et al. (2021) [16]	USA	292	196	96	Phase III, randomized, double-blind, placebo-controlled, two-arm trial	Adolescents (12-17 years)	400-600 mg ER	Six weeks	ADHD-RS-5	600 mg dose was not statistically significant vs placebo. 400 mg dose showed greater reduction in ADHD symptoms vs placebo, but was not formally declared superior due to hierarchical testing.

Risk of Bias Assessment

Risk of bias was assessed using the NIH Study Quality Assessment Tool [12]. The quality assessment was conducted independently by two reviewers (S.C. and C.R.). No disagreements occurred between reviewers. Scoring was conducted by awarding one point for “yes” responses and zero points for “no” responses to the questions. An article was deemed “poor” if it was awarded from zero to four points, fair if it was awarded from five to nine points, and “good” if it was awarded 10-14 points. Results from the risk of bias assessment are presented in Table 2. All three studies were rated as “good” quality. Each study received an “unclear” in the allocation concealment category, as specific randomization measures were not explicitly reported.

**Table 2:** Risk of bias quality assessment using the NIH Study Quality Assessment Tool Items 1-14 represent the 14 criteria questions used in the NIH Quality Assessment of Controlled Intervention Studies, a bias assessment tool that measures the methodological quality of each study. A "yes" or "no" answer is provided for each question. Each number represents the following questions: 1. Was the study described as a randomized clinical trial? 2. Was the method of randomization adequate? 3. Was the treatment allocation concealed? 4. Were the study participants and providers blinded to treatment group assignment? 5. Were the people assessing the outcomes blinded to the participants' group assignments? 6. Were the groups similar at baseline on important characteristics that could affect outcomes? 7. Was the overall drop-out rate from the study at endpoint 20% or lower than the number allocated to treatment? 8. Was the differential drop-out rate at endpoint 15 percentage points or lower? 9. Was there high adherence to the intervention protocols for each treatment group? 10. Were other interventions avoided or similar in the groups? 11. Were outcomes assessed using valid and reliable measures implemented consistently across all study participants? 12. Did the author report that the sample size was sufficiently large to be able to detect a difference in the main outcome between groups with at least 80% power? 13. Were outcomes reported or subgroups analyzed pre-specified? 14. Were all randomized participants analyzed in the group to which they were originally assigned? N: no; Y: yes; NIH: National Institute of Health

Study	Bias ruling	Total score (out of 14)	1	2	3	4	5	6	7	8	9	10	11	12	13	14
Nasser et al. (2022) [14]	Good	13	Y	Y	Unclear	Y	Y	Y	Y	Y	Y	Y	Y	Y	Y	Y
Nasser et al. (2021) [15]	Good	13	Y	Y	Unclear	Y	Y	Y	Y	Y	Y	Y	Y	Y	Y	Y
Nasser et al. (2021) [16]	Good	13	Y	Y	Unclear	Y	Y	Y	Y	Y	Y	Y	Y	Y	Y	Y

Findings

Efficacy Outcomes

**Figure 2:**
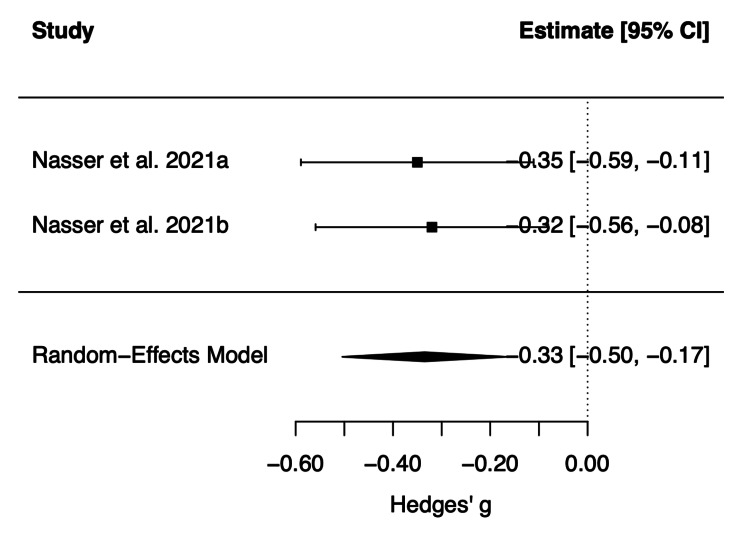
Forest plot of standardized mean differences (Hedges’ g) comparing viloxazine extended-release (ER) with placebo in adolescents with attention-deficit/hyperactivity disorder (ADHD) A random-effects model was used to pool effect estimates. Squares represent individual study effect estimates with horizontal lines indicating 95% CIs. The diamond represents the pooled effect estimate. Studies included in the analysis were Nasser et al. (2021a) [15] and Nasser et al. (2021b) [16].

Adolescent population: A random-effects meta-analysis was conducted to evaluate the efficacy of viloxazine ER in adolescents with ADHD. Of the 593 participants across both studies, 393 received viloxazine ER and 200 received placebo. Study level effect sizes were calculated using change from baseline in ADHD symptom severity as assessed with the ADHD-RS-5 scale compared with placebo. The results of the random-effects model comparing changes in viloxazine ER and placebo groups are presented below (Figure 2).

Both studies demonstrated reductions in ADHD symptom severity compared to placebo as measured by the ADHD-RS-5. In Nasser et al. (2021b), only the 400 mg/day dose demonstrated statistical significance vs placebo based on the pre-specified sequential testing procedure. The 600 mg/day dose did not meet significance criteria, possibly due to an unusually high response to placebo [16]. Despite this, pooled analysis demonstrated a moderate improvement in ADHD symptom severity, favoring viloxazine ER over placebo (Hedges’ g = -0.33, 95% CI: -0.50 to -0.16). No between-study heterogeneity was observed (I² = 0%).

Adult Population

One RCT evaluating viloxazine ER in adults met the inclusion criteria. In this trial, viloxazine ER was associated with a statistically significant improvement in ADHD symptoms vs placebo. This improvement was measured using the AISRS from baseline to study endpoint (six weeks). The viloxazine ER group demonstrated greater mean improvement based on this scale in comparison to placebo. Reported adverse events were generally mild to moderate and consistent with the established safety profile of viloxazine ER, including nausea, insomnia, and headache. Adult outcomes are summarized descriptively in the following section below. 

Discussion

This systematic review and meta-analysis evaluated the efficacy and tolerability of viloxazine ER for the treatment of ADHD in adolescent and adult populations. By synthesizing data from eligible RCTs, this analysis demonstrates that viloxazine ER is associated with a statistically significant reduction in ADHD symptoms compared with placebo in adolescents. The pooled effect size was modest and consistent across both studies. No statistical heterogeneity was observed. 

The observed pooled effect size (Hedges’ g = -0.3) was similar to that reported for other non-stimulant ADHD medications such as atomoxetine and guanfacine [17,18]. It should be noted, however, that the effect size is not as dramatic as the effect sizes reported for stimulants [19]. Yet non-stimulant medications play an important role in patients who cannot tolerate the side effects of stimulants and provide the benefit of an increased safety profile [5]. These findings support viloxazine ER as an important alternative medication or adjunctive treatment option for reducing ADHD symptoms.

Adolescence is a distinct developmental period characterized by unique hormonal, environmental, and psychosocial changes. These changes may influence symptom presentation and affect treatment response, highlighting the need for evidence to be synthesized specifically within this population [7]. Focusing specifically on the adolescent population provides insight into a population that could benefit from tailored and individualized treatment approaches. 

Adult data on viloxazine ER remain limited. Available evidence is primarily derived from one RCT, including a total of 374 patients. Despite demonstration of statistically significant improvement in ADHD symptoms among adults compared with placebo, the limited number of studies prevented quantitative pooling [14]. This underscores an important gap in current literature. Additional RCTs evaluating the efficacy of viloxazine ER in adult populations should be encouraged.

Safety and Tolerability

Viloxazine ER was generally well tolerated in both adolescent and adult populations, with low discontinuation rates reported across the included studies [14-16]. Most reported adverse effects were mild to moderate in severity and were consistent with the pharmacologic profile of non-stimulant ADHD medications. Frequently reported adverse events included insomnia, somnolence, decreased appetite, dry mouth, and fatigue. 

Discontinuation rates were low and comparable to the discontinuation rates observed in placebo groups. Severe adverse events were uncommon. In the adolescent studies, no dose-dependent increase in adverse events was identified. In the included adult study, the safety profile was comparable to placebo. Overall, viloxazine ER demonstrated a favorable tolerability and safety profile in adolescents and adults with ADHD. A summary of commonly reported adverse events and discontinuation rates across the included studies is presented in Table 3.

**Table 3:** Summary of common adverse events VLX-ER: Viloxazine Extended-Release; AEs: adverse events

Study	Population	Most common adverse events (VLX-ER)	Discontinuation due to AEs
Nasser et al. (2022) [14]	Adults	Insomnia, fatigue, nausea, decreased appetite, dry mouth, headache, and constipation	Low; 9.0%
Nasser et al. (2021) [15]	Adolescents	Somnolence, headache, decreased appetite, nausea, and fatigue	Low; 2.9%
Nasser et al. (2021) [16]	Adolescents	Somnolence, fatigue, headache, nausea, and decreased appetite	Low; 4.5%

Limitations

The number of RCTs eligible for quantitative meta-analysis was small, which limited statistical power and formal assessment of publication bias. Even though there was no statistical heterogeneity, the pooled estimates should be interpreted with caution. 

Another consideration to take into account is that the included studies were conducted by overlapping investigator groups. Although these studies utilized randomized, double-blinded, placebo-controlled designs and were assessed as having low risk of bias, publication bias cannot be entirely excluded [20].

Adult outcomes were not pooled quantitatively because only one adult study met the inclusion criteria. Consequently, conclusions regarding efficacy in adults are based on descriptive synthesis rather than quantitative analysis. 

The included trials evaluated short-term efficacy outcomes over six-week periods. As a result, long-term safety and effectiveness were not evaluated, which should be further investigated in future studies. Despite these limitations, this study provides a focused synthesis of the available randomized evidence supporting the usage of viloxazine ER in adolescents and adults with ADHD.

## Conclusions

This analysis highlights the role of the emerging non-stimulant medication viloxazine (Qelbree) as an alternative treatment option for ADHD in adolescent and adult populations. This study found statistically significant, moderate improvements in ADHD symptoms among adolescents treated with viloxazine ER compared with placebo. The pooled effect demonstrated no evidence of statistical heterogeneity, supporting the consistency of treatment effects. Additionally, viloxazine ER was generally well tolerated with an adverse event profile similar to placebo. 

Although evidence for viloxazine ER in adult populations remains limited, available data suggest benefit and underscore the need for additional research in this age range. The findings presented here support viloxazine ER as a clinically useful non-stimulant treatment option for reducing ADHD symptoms in adolescents, particularly for patients in whom stimulant pharmacotherapy is contraindicated or undesirable. Future studies should prioritize long-term outcomes and efficacy across broader age groups.
